# Efforts to Address the Burden of Non-Communicable Diseases Need Local Evidence and Shared Lessons from High-Burden Countries

**DOI:** 10.5334/aogh.4118

**Published:** 2023-11-17

**Authors:** Jackline E. Ngowi, Castory Munishi, Harrieth P. Ndumwa, Belinda J. Njiro, Davis E. Amani, Erick A. Mboya, Doreen Mloka, Amani I. Kikula, Emmanuel Balandya, Paschal Ruggajo, Anna T. Kessy, Emilia Kitambala, Ntuli Kapologwe, James T. Kengia, James Kiologwe, Omary Ubuguyu, Bakari Salum, Appolinary Kamuhabwa, Kaushik Ramaiya, Bruno F. Sunguya

**Affiliations:** 1 Muhimbili University of Health and Allied Sciences, 9 United Nations Road, Upanga West P O Box 65001, Dar es salaam, Tanzania; 2 Ministry of Health, P O Box 743 Dodoma, Tanzania; 3 President’s Office Regional Administration and Local Government, P O Box 1923 Dodoma, Tanzania; 4 Tanzania Non-Communicable Diseases Alliance, P O Box 65201 Dar es salaam, Tanzania; 5 Tanzania Diabetes Association, P O Box 65201 Dar es salaam, Tanzania; 6 Shree Hindu Mandal Hospital, P O Box 581 Dar es salaam, Tanzania

**Keywords:** non-communicable diseases (NCDs), epidemiological transition, advocacy, Tanzania

The burden of non-communicable diseases (NCD) is increasing at an unprecedented rate globally [1,2]. The surge in recent years strains the already weakened health system in low and middle-income countries (LMICs). Such epidemiological transition in most resource-constrained countries occurs under the constrained health system struggling to contain the persistent burden of communicable diseases [3,4] deaths arising from LMICs, calling for immediate locally driven interventions, but with informed policies and intervention to counteract the drivers of such burden.

Although the standard drivers for NCDs are globally known, namely, tobacco use, physical inactivity, the harmful use of alcohol, and unhealthy diets [5]. These risk factors vary from one country and region to another [6,7]. Therefore, the globally and World Health Organization’s (WHO) recognized best buys may not be adequate if not tailored to the locally determined risk factors, local epidemiology, context, and matching the available resources. These differences and lack of comprehensive knowledge of NCD’s risk factors call for country-led research to understand the local epidemiology, use evidence-based interventions, and apply multi-stakeholder approaches to address such a multi-faceted burden [8,9]. Countries need ownership and use of local resources, adequate healthcare financing for chronic diseases, and community-based initiatives that work within the same contexts to ensure sustainability [10,11].

This series presents efforts by countries facing epidemiological transition with the surge of NCDs amid the persistent burden of communicable diseases [12,13,14,15]. The majority of these countries are in low and middle-income brackets [16,17,18,19]. Such countries are making various efforts to contain the unprecedented burden, drawing several lessons from one another in reaching epidemiological control. Some of the initiatives presented in this series are unique and can be adopted with modifications to suit other countries or regions with similar contexts.

Uniquely, three of the papers presented in this series are from Tanzania. The country has a persistent burden of traditional communicable diseases such as HIV at a national prevalence of 4.7% [20], Malaria at a national prevalence of 9% [21], and tuberculosis with an incidence rate of 222 per 100000 of the population [22], maternal and child health challenges with maternal mortality 524 deaths per 100000 [23]. Under such heavy burden of communicable diseases, Tanzania is facing a fact growing burden of NCDs, accounting for 41% of the Disability Adjusted Life Years (DALYs) [24], and NCDs alone accounting for more than 31–34% of premature deaths [25,26]. The burden of NCDs aligns with nutritional, economic, and demographical transitions [27]. Whereas the common risk factors such as unhealthy dietary habits, overweight and obesity, tobacco intake, and excessive alcohol consumption are associated with the big four NCDs in the country, namely: cardiovascular diseases, cancers, chronic respiratory diseases, and diabetes; they account for only 40% of the known risk factors [24]. Furthermore, the burdens of other NCDs, such as road traffic injuries, mental health disorders, and congenital conditions, are also rising and equally demand immediate attention. The confluence of the previous and persistent burden of childhood under nutrition in Tanzania [28,29] may also attribute to the unknown risk factors found elsewhere [30,31]. The high burden of maternal under-nutrition [32] may be behind poor birth outcomes, such as low birth weight, which may in turn explain the rising NCD burden in terms of morbidity and mortality in the country [33]. Understanding the local contextual factors driving the NCDs burden is therefore crucial for any meaningful success.

Strengthening the health system has been a hallmark in efforts to address the local burden in Tanzania [24]. The Government of Tanzania, through the Ministry of Health (MoH), has been working with the Tanzania Non-communicable Diseases Alliance (TANCDA) and Tanzania Diabetes Association (TDA), and other national and international organizations with interest in NCDs, pioneered the development of the National Strategic Plan for NCDs launched in 2009 [34]. The collaboration between government and non-government stakeholders resulted in the development of the National NCDs Control and Prevention Program in 2019 to coordinate the national preventive, curative, and rehabilitative efforts and align the stakeholders [35].

Although the burden is still rising on all NCDs in Tanzania [36], the country is on the right track in NCDs response [37]. Tanzania is far away from having a transformative and resilient health system to cope with the rising cost of health care from such chronic conditions [38,39,40]. The strategies’ implementation and stakeholder collaboration should also emphasize prevention and control measures. The focus on NCD prevention and control heralds the start of a new era in providing affordable health services while maintaining equity and quality health services for those affected by chronic diseases and disabilities for the rest of their lives [40,41].

Through the coordinated efforts, the national NCDs Control and Prevention Program, the national NCDs week was inaugurated to commemorate various efforts addressing the NCDs burden in the country. In this particular week, the MoH coordinates the national NCDs conference serving as a platform to convene scientists in Tanzania and beyond to discuss the burden and national response and innovations to address the new challenges. The NCDs week has advocacy activities, school health promotion, physical activities, and NCDs screening. The week attracts national and civil organizations leaders, a sign of commitment to addressing the burden of NCDs. As explained in these papers, such efforts may inform other countries with similar burdens or contexts in setting up or strengthening their national and sub-countries’ response against the rising burden of NCDs.
